# FixPred: a resource for correction of erroneous protein sequences

**DOI:** 10.1093/database/bau032

**Published:** 2014-04-04

**Authors:** Alinda Nagy, László Patthy

**Affiliations:** Institute of Enzymology, Research Centre for Natural Sciences, Hungarian Academy of Sciences, H-1113 Budapest, Hungary

## Abstract

Protein databases are heavily contaminated with erroneous (mispredicted, abnormal and incomplete) sequences and these erroneous data significantly distort the conclusions drawn from genome-scale protein sequence analyses. In our earlier work we described the MisPred resource that serves to identify erroneous sequences; here we present the FixPred computational pipeline that automatically corrects sequences identified by MisPred as erroneous. The current version of the associated FixPred database contains corrected UniProtKB/Swiss-Prot and NCBI/RefSeq sequences from *Homo sapiens*, *Mus musculus*, *Rattus norvegicus*, *Monodelphis domestica*, *Gallus gallus*, *Xenopus tropicalis*, *Danio rerio*, *Fugu rubripes*, *Ciona intestinalis*, *Branchostoma floridae*, *Drosophila melanogaster* and *Caenorhabditis elegans*; future releases of the FixPred database will include corrected sequences of additional Metazoan species. The FixPred computational pipeline and database (http://www.fixpred.com) are easily accessible through a simple web interface coupled to a powerful query engine and a standard web service. The content is completely or partially downloadable in a variety of formats.

**Database URL:** http://www.fixpred.com

## Introduction

Medical sciences, drug development, agriculture and biotechnology rely increasingly on information originating from genome projects. One of the most crucial steps in the interpretation of genome sequences is the computational identification of protein-coding genes and prediction of their structure, the success of all subsequent steps of protein research exploiting genomic sequences depends on the quality of these predictions.

Despite significant improvements in gene-prediction technologies, prediction of the structure of protein-coding genes of higher eukaryotes remains a difficult task; according to current estimates, the structure of only ∼60% of predicted human genes is correct (1, 2). Because erroneous data generated by misprediction are carried forward en masse to other databases and biological conclusions are drawn from the erroneous data, this may significantly distort the results of genome-scale protein sequence analyses (3–9).

To solve this problem—in our earlier MisPred project—we have developed a method that helps decide whether the structure of an in silico predicted or experimentally supported protein-coding gene is erroneous (mispredicted, abnormal or incomplete). The MisPred approach is based on the principle that the structure of a protein-coding gene is likely to be erroneous if some of the features of the protein-coding gene or the predicted protein conflict with some of the dogmas about protein-coding genes and proteins (3, 10). The current version of the MisPred computational pipeline uses 11 distinct tools to identify erroneous sequences affected by different types of errors (10).

Tool 1. Conflict between the presence of obligatory extracellular protein domain(s) (http://www.mispred.com/table1to3) in a protein and the absence of appropriate sequence signals that could direct the extracellular domain(s) into the extracellular space.
Tool 2. Conflict between the presence of obligatory extracellular and cytoplasmic domains (http://www.mispred.com/table1to3) in a protein and the absence of transmembrane helix(ces).
Tool 3. Co-occurrence of obligatory extracellular and nuclear domains (http://www.mispred.com/table1to3) in a protein.
Tool 4. Domain size deviation.
Tool 5. Inter-chromosomal chimeric proteins.
Tool 6. Conflict between the presence of secretory signal peptide and obligatory cytoplasmic protein domains (http://www.mispred.com/table1to3) in a protein and the absence of transmembrane segments.
Tool 7. Conflict between the presence of GPI-anchor in a protein and the absence of secretory signal peptide.
Tool 8. Co-occurrence of GPI-anchor and obligatory cytoplasmic protein domains (http://www.mispred.com/table1to3) in a protein.
Tool 9. Co-occurrence of GPI-anchor and obligatory nuclear protein domains (http://www.mispred.com/table1to3) in a protein.
Tool 10. Co-occurrence of GPI-anchor and transmembrane segments in a protein.
Tool 11. Domain architecture deviation.

By identifying mispredicted protein sequences, the MisPred pipeline serves to inform the creators of the predictive algorithms of the reliability of predictions, thereby assisting the improvement of gene prediction technologies. The MisPred approach is also useful for the identification of abnormal and incomplete proteins: with the help of the MisPred tools we could show that a significant proportion of alternatively spliced mRNAs do not encode viable proteins (11) and that many, allegedly full-length cDNAs are in fact incomplete (3).

Elimination of erroneous entries from public databases is of crucial importance because it might protect users from drawing erroneous conclusions based on erroneous data, but it is even more important to correct erroneous sequences. Given the flood of erroneous data from genome projects, there is an increasing need for computational tools that perform these corrections on a mass scale.

The main objective of our FixPred project is to develop the FixPred pipeline for the automatic correction of sequences identified by MisPred as erroneous and to construct the FixPred database in which corrected versions of erroneous sequences are deposited.

## The FixPred pipeline

We have designed the FixPred pipeline to correct abnormal, incomplete and mispredicted proteins primarily from Metazoan species. The rationale of the FixPred approach is that an erroneous sequence (identified as such by MisPred) is judged to be corrected if the correction eliminates the error(s) identified by MisPred.

Note that MisPred does not only state that a sequence is erroneous but also identifies the ‘type of error’, thereby pinpointing the ‘location of the error’ (Supplementary Figure S1). For example, if a protein is identified as erroneous by MisPred tool 1 (i.e. the protein contains domains that occur exclusively in the extracellular space but lacks a secretory signal peptide or signal anchor sequence that could direct the domain into the extracellular space), then we know that the error affects the N-terminal part of the sequence and this error may be corrected by identifying the missing secretory signal peptide or signal anchor sequence (Supplementary Figure S1A). Similarly, if a protein is identified as erroneous by MisPred tool 2 (i.e. it contains both extracellular and cytoplasmic protein domains but lacks transmembrane helices that pass through the membrane), then we know that the error is located internally, between the extracellular and cytoplasmic domains of the protein, and this error may be corrected by identifying the missing transmembrane helix (Supplementary Figure S1B).

There are multiple ways to correct an erroneous protein sequence deposited in a database: (i) the correct sequence may already exist in other protein databases; (ii) protein, cDNA and EST databases may contain sufficient amount of information to assemble a corrected version of the erroneous sequence; (iii) a corrected version of the protein may be predicted by subjecting the genome sequence to computational gene predictions.

**Figure 1.**
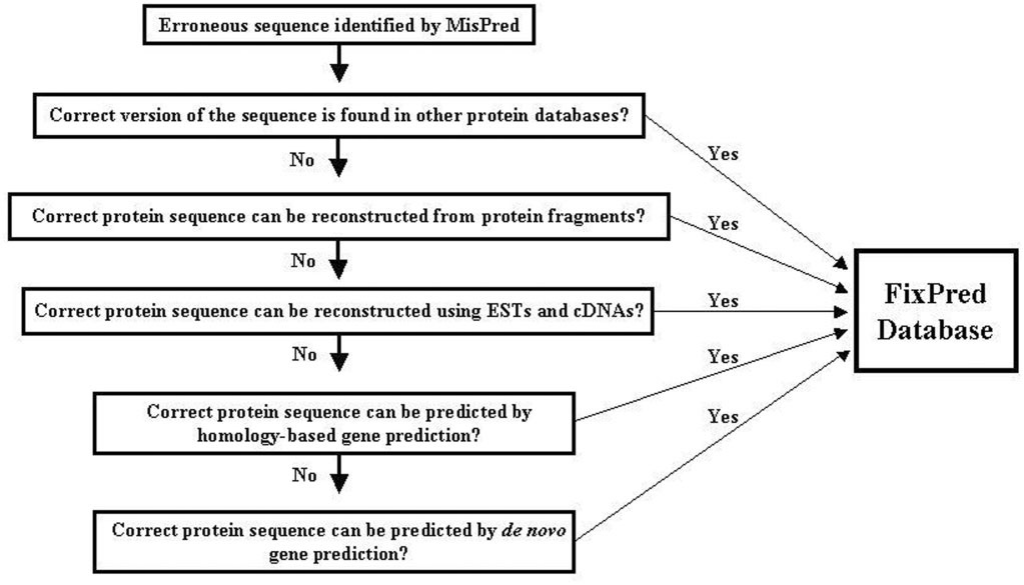
Flow chart of the FixPred pipeline.

The FixPred pipeline attempts to correct erroneous sequences in several steps, starting with the simplest solutions (finding experimental evidence for the correct sequence version in existing protein or cDNA and EST databases), progressing to more time-consuming gene-predictions. The FixPred software package corrects erroneous sequences according to a dichotomic decision tree (see flow chart in Figure 1):

**Step 1. MisPred identifies a sequence as erroneous.** The sequence is used as input of the FixPred pipeline. Because the false-positive rates of some MisPred tools are relatively high (3, 4, 10), users are advised to subject sequences identified by MisPred as suspicious to additional analyses to decide whether the protein is truly erroneous or a false positive before they subject it to sequence correction by the FixPred pipeline.
**Step 2. Search for a correct version of the erroneous sequence in other protein databases.** If these searches find a correct version (that is not affected by the error detected by MisPred), then the corrected version is deposited in the FixPred database and the correction procedure is terminated. If these searches fail to find a correct version, then the erroneous sequence is used as input in Step 3.
**Step 3. Reconstruction of a corrected protein sequence using overlapping protein fragments.** If sequence searches in Step 2 identified fragments that overlap with the erroneous sequence but differ from it in the region affected by the error, then FixPred uses the overlapping fragments to reconstruct sequences (Supplementary Figure S2). If these reconstructions correct the error identified by MisPred, then the corrected sequence is deposited in the FixPred database and the correction procedure is terminated. If this step fails to reconstruct a corrected version, then the erroneous sequence is used as input in Step 4.
**Step 4. Reconstruction of a corrected protein sequence using overlapping ESTs or cDNAs.** ESTs/cDNAs that overlap with the erroneous sequence but differ from it in the region affected by the error are used to reconstruct sequences. If these reconstructions correct the error identified by MisPred, then the corrected sequence is deposited in the FixPred database and the correction procedure is terminated. If this step fails to reconstruct a correct version then the erroneous sequence is used as input in Step 5.
**Step 5. Homology-based prediction of a corrected version of the erroneous sequence using genomic sequence.** The erroneous sequence is used to search for non-erroneous homologs from the same species (paralogs) and from other species (orthologs and paralogs). The genomic region that encodes the erroneous sequence is subjected to homology-based gene prediction, using the closest non-erroneous homologs. If the predictions include sequences (or sequence fragments) that are not affected by the original error then these are used to correct the erroneous sequence. As predictions that correct the original error may introduce errors elsewhere, only the corrected region is used in the reconstruction of the corrected version. If these reconstructions correct the error identified by MisPred, then the corrected sequence is deposited in the FixPred database and the correction procedure is terminated. If this step fails to reconstruct a corrected version, then the erroneous sequence is used as input in Step 6.
**Step 6. De novo prediction of a corrected version of the erroneous sequence using genomic sequence.** The genomic region that encodes the erroneous sequence is analyzed with tools of de novo gene prediction. If the predictions include sequences (or sequence fragments) that are not affected by the original error, then these are used to correct the erroneous sequence. As predictions that correct the original error may introduce errors elsewhere, only the corrected region is used in the reconstruction of the correct version. If these reconstructions correct the error identified by MisPred, then the corrected sequence is deposited in the FixPred database and the correction procedure is terminated.

There are several arguments in favor of the decision tree outlined above. Although there is no guarantee that Steps 2, 3 and 4 succeed in finding experimental evidence for a correct version, this is the simplest and most straightforward way of error correction. If these steps fail, FixPred proceeds to Steps 5 and 6 that are more time-consuming but have a chance to succeed if the genome sequence is known.

The FixPred pipeline exploits public databases and a variety of standard software:

**Steps 2 and 3.** In these steps, the pipeline uses the erroneous sequence as a query to search the UniProtKB/Swiss-Prot, UniProtKB/TrEMBL (12), EnsEMBL (13) and NCBI/RefSeq (14, 15) protein databases with blastp (16) limiting the search to the same species as the source of the query sequence. FixPred selects protein sequences that are >98% identical (allowance for sequencing errors and polymorphisms) with the query sequence over >25 residues, and these sequences are analyzed by the same MisPred tools as the ones that identified the query sequence as being suspicious. Sequences that are not affected by the errors that affected the query sequence are concluded to be the correct versions of the erroneous sequence. If the analysis finds only protein fragments that overlap with the query sequence but differ from it in the region affected by the error, then the erroneous sequence is corrected with these overlapping fragments, eliminating the error through the assembly of the fragments (Supplementary Figure S2).
**Step 4.** In this step, key ESTs and cDNAs are identified with the query sequence or with the closest non-erroneous homologs of the query sequence.

First, the erroneous sequence is used as query to search EST and cDNA databases (15) with tblastn (16), limiting the search to the species from which the erroneous sequence originates. The program selects sequences that are >80% identical (allowance for sequencing errors and polymorphisms) with the query sequence over >25 amino acid residues. EST or cDNA sequences thus selected are translated in the reading frame corresponding to the query sequence using Transeq (17). If these analyses find fragments that overlap with the erroneous sequence but differ from it in the region affected by the error, then the erroneous sequence is corrected with these overlapping sequences, eliminating the error by the assembly of the fragments (Supplementary Figure S3).

If the search with the erroneous sequence failed to find ESTs/cDNAs satisfying these criteria (lack of extensive overlap over >25 residues), then the closest non-erroneous homologs are used to identify key ESTs/cDNAs that may originate from the region affected by the error. To find non-erroneous homologs, FixPred uses the erroneous sequence as a query to search the UniProtKB/Swiss-Prot, UniProtKB/TrEMBL, EnsEMBL and NCBI/RefSeq protein databases using blastp, extending the search to other Metazoan species. Fifty homologs with the lowest E-values (E-value <10^−^^25^) are analyzed by MisPred, and FixPred selects sequences that do not show signs of the same type of error as the query (Supplementary Figure S4).

The closest non-erroneous homologs with the highest percent identity are used to identify ESTs/cDNAs that might be used for the correction of the erroneous sequence. To achieve this, the sequence region that distinguishes the correct homolog from the erroneous sequence, plus 30 amino acid residues of the regions where their sequences overlap, is used as query to search EST and cDNA databases with tblastn, limiting the search to the species from which the erroneous sequence originates. FixPred selects homologous EST or cDNA sequences that are >50% identical (at the amino acid level) with the non-erroneous homolog over >25 amino acid residues. Selected EST sequences are translated in the reading frame corresponding to the query sequence using Transeq (17). If these analyses find fragments that are identical with the erroneous sequence over >10 amino acid residues but differ from it in the region affected by the error, then the erroneous sequence is corrected with these overlapping sequences, eliminating the error by the assembly of the fragments (Supplementary Figure S4).

**Step 5.** In this step, the closest non-erroneous homologs (with highest per cent identity) are used to predict the correct version of the erroneous sequence from genomic sequence. First, the erroneous sequence is used as a query to identify the genomic region containing the gene for the protein using tblastn, and then the sequence of the closest non-erroneous homolog is used to find evidence for the parts that distinguish the erroneous and non-erroneous sequences. If the latter search finds evidence for exons resolving the error, then the genomic region encoding the query protein is extended to include regions that are expected to encode the correct exons. The genomic region thus selected is subjected to gene prediction with GeneWise (18), using the sequence of the non-erroneous homolog as input. Predicted protein sequences that resolve the original error are used to correct the erroneous sequence. Because the prediction that corrects the original error may introduce errors elsewhere, only the corrected region is used in the reconstruction.
**Step 6.** In this step, the genomic region encoding the erroneous sequence is analyzed with de novo gene-finding programs GeneScan (19) and Augustus (20). Predicted protein sequences that resolve the original error are used to correct the erroneous sequence. Because the prediction that corrects the original error may introduce errors elsewhere, only the corrected region is used in the reconstruction.

## The FixPred database

The current version of the FixPred database contains 1462 corrected UniProtKB/Swiss-Prot and NCBI/RefSeq sequences from *Homo sapiens*, *Mus musculus*, *Rattus norvegicus*, *Monodelphis domestica*, *Gallus gallus*, *Xenopus tropicalis*, *Danio rerio*, *Fugu rubripes*, *Ciona intestinalis*, *Branchostoma floridae*, *Drosophila melanogaster* and *Caenorhabditis elegans*; future releases of the FixPred database will include corrected sequences of additional Metazoan species.

FixPred entries contain the FixPred ID, the species name, the corrected protein sequence (in FASTA format), the type of evidence on protein existence and the date of publication. The FixPred database contains two types of corrected sequences. Sequences with FXP identifiers denote sequences that were corrected automatically by the FixPred pipeline and checked manually by an expert. The FIXEXP identifiers denote sequences where the corrected sequence was validated experimentally by cDNA cloning.

The ‘Protein existence’ field indicates the type of evidence that supports the existence of the protein. FixPred lists four types of evidences for the existence of a protein: (i) evidence at protein level; (ii) evidence at transcript level; (iii) evidence at EST level; and (iv) evidence at genome level. Only the highest or most reliable level of supporting evidence for the existence of a protein is displayed for each entry. For example, if the existence of a protein is supported by both ESTs and cDNAs, then the ‘Protein existence’ field indicates ‘Evidence at transcript level’.

**Figure 2.**
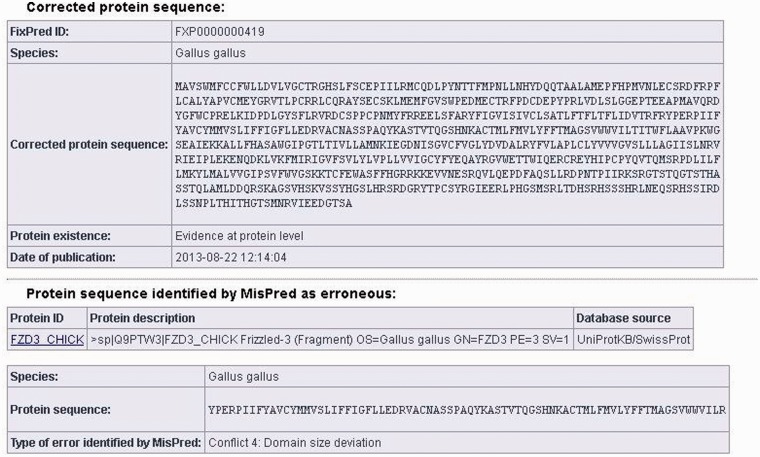
Screen shot of an entry of the FixPred database. The figure shows the corrected version (upper part) of an erroneous protein sequence of *G. gallus*, deposited in the UniProtKB/SwissProt database with the protein ID: FZD3_CHICK (lower part). The FZD3_CHICK protein was identified as erroneous by MisPred tool 4 (domain size deviation) because it contains only a fragment of the Frizzled (PF01534) domain. The erroneous protein was corrected by the FixPred pipeline in Step 2 by identifying a full-length version of the frizzled-3 precursor (NP_001258869.1).

The data sheets of the corrected protein sequences also contain information about the original protein sequences identified by MisPred as erroneous: the protein ID, the protein description, the database source, the species name, the erroneous protein sequence (in FASTA format) and the type of sequence error(s) identified by MisPred are provided. (It should be noted that if the same erroneous sequence was deposited multiple times in the same or different databases, several protein IDs and database sources are listed.) A typical example of a FixPred entry is shown in Figure 2.

## Database statistics

The 1462 corrected sequences of the current version of the FixPred database were generated by the FixPred pipeline through the analysis of 8118 erroneous UniProtKB/Swiss-Prot and NCBI/RefSeq entries from 12 Metazoan species. It must be pointed out that this ratio (0.18) of corrected and erroneous sequences does not equal the rate of correction. Frequently, the same erroneous sequence is deposited multiple times in the same or different databases with different protein IDs, and because their correction by the FixPred pipeline yields the same sequence, the rate of correction may be underestimated. Another source of underestimation is that several different erroneous versions of the same protein may be present in public databases and their correction may yield the same corrected sequence. Conversely, in the case of fusion proteins (e.g. inter- and intra-chromosomal chimeras), correction of a single erroneous sequence is expected to yield two corrected sequences through the separation of the constituent proteins.

Despite these caveats, it is worth noting that the apparent rate of correction is lower in the case of UniProtKB/Swiss-Prot entries than in the case of RefSeq sequences: a total of 61 corrected sequences were obtained through the analysis of 456 Swiss-Prot sequences (13.4%), whereas analysis of 7662 NCBI/RefSeq entries resulted in 1418 corrected sequences (18.5%). This observation is in harmony with the higher quality of the Swiss-Prot database and the fact that the NCBI/RefSeq database contains a relatively high proportion of mispredicted sequences.

With respect to the type of sequence error, extracellular proteins lacking secretory signal peptides and protein sequences affected by domain size deviation constitute the largest proportion of erroneous sequences and the apparent rates of their correction are also among the highest (23.5 and 29.1%, see Table 1), making MisPred Tools 1 and 4 the most valuable constituents of the MisPred and FixPred pipelines. Co-occurrence of extracellular and nuclear domains in a protein is a relatively rare type of error (identified by MisPred tool 3) but its rate of correction is very high (Table 1) even if we take into account the fact that correction of an erroneous fusion sequence is expected to yield two corrected sequences (see Supplementary Figure S1C).

**Table 1.** Rate of correction of different types of sequence errors

Error type identified^a^	Erroneous sequences	Corrected sequences	Apparent rate of correction (%)
MisPred tool 1	3394	799	23.5
MisPred tool 2	10	2	20.0
MisPred tool 3	12	16	133.3^b^
MisPred tool 4	2033	592	29.1
MisPred tool 5	890	36	4.0
MisPred tool 6	916	4	0.4
MisPred tool 7	479	32	6.7
MisPred tool 8	50	0	0.0
MisPred tool 9	3	0	0.0
MisPred tool 10	331	3	0.9

^a^Erroneous sequences identified by MisPred tool 11 and corrected by the FixPred pipeline are not yet deposited in the FixPred database. These data will be released in the next update of FixPred.

^b^In the case of MisPred tool 3, correction of an erroneous sequence containing both nuclear and extracellular domains is expected to yield two corrected sequences (Supplementary Figure S1C).

The apparent rate of correction shows significant variation with respect to the species from which the erroneous sequence originates. As shown in Table 2, the highest rate of correction is observed in the case of *H. sapiens* sequences, whereas the lowest rates are observed in the case of *B. floridae*, *C. intestinalis* and *D. rerio*. The most plausible explanation for these differences in rate of correction is that they reflect differences in the availability of experimental information on protein sequences (full length proteins in other databases, protein fragments, cDNAs etc.) that facilitate the correction process through Steps 2 and 3 of the FixPred pipeline. This interpretation is supported by our observation that the highest proportion of the corrections was completed in Steps 2 (56.2%) and 3 (37.0%), i.e. a correct version of the erroneous sequence is present in other databases or can be reconstructed from fragments (Table 3). Note that in the ‘Sequences corrected’ column of Table 3 the sum of sequences corrected in Steps 2–6 (1532) exceeds the total number of sequences corrected (1462). The primary source for this difference is that the same erroneous sequence is deposited multiple times in the same or different databases with different protein IDs but their correction by the FixPred pipeline yields the same sequence.

**Table 2.** Rate of correction of erroneous sequences of different metazoan species

Species	Erroneous sequences	Corrected sequences	Apparent rate of correction (%)
*H. sapiens*	941	331	35.2
*M. musculus*	455	106	23.3
*R. norvegicus*	704	178	25.3
*M. domestica*	434	93	21.4
*G. gallus*	458	118	25.8
*X. tropicalis*	547	176	32.2
*D. rerio*	1376	180	13.1
*F. rubripes*	507	97	19.1
*B. floridae*	1753	46	2.6
*C. intestinalis*	391	28	7.2
*C. elegans*	215	49	22.8
*D. melanogaster*	337	60	17.8

**Table 3.** Correction of erroneous proteins in different steps of the FixPred pipeline (see Figure 1)

Steps	Sequences analyzed	Sequences corrected	Proportion corrected	Percent of total correction
Total	8118	1462	0.18	100
Step 2	8118	822	0.10	56.2
Step 3	7296	541	0.07	37.0
Step 4	6755	75	0.01	5.1
Step 5	6680	73	0.01	5.0
Step 6	6607	21	0.00	1.4

## Database implementation

The database is built on an Apache HTTP Server 2.2.6 with Oracle Database 11g Server. The front end was developed using play! 1.2.4 (http://www.playframework.org) framework with HTML and JAVA script, and the back end was developed using Oracle Database 11g Server, a relational database management system. All common gateway interface and database interfacing scripts were written in Java programming language.

## Web interface

The FixPred web interface is designed to explain the goals and principles of the MisPred and FixPred projects (web page ABOUT FIXPRED) and to allow the user to rapidly query the complete database (web page SEARCH FIXPRED) or to use the various FixPred tools to correct sequences identified as erroneous by MisPred (web page CORRECT YOUR SEQUENCE).

### Search tools

FixPred provides three search options on the ‘SEARCH FIXPRED’ page: the simple, the advanced and the similarity search options. The Simple search option allows users to query any field of the database entries (protein ID in the source database, FixPred ID of the corrected sequence, protein description, database source, species name, type of sequence error identified by MisPred). Under the Advanced search option, users can combine queries of the different fields using the AND, OR and NOT operators.

The ‘Find best match of your sequence in FixPred database by similarity’ feature of SEARCH FIXPRED is meant to find the corrected version of an erroneous query sequence. The idea behind this feature is to spare the time of sequence correction if the correct sequence is already deposited in the FixPred database.

On initiating the search, the IDs of all protein sequences matching the criteria of the search are displayed. In the case of similarity searches, the alignments also may be displayed. For each protein sequence retrieved (see Figure 2), a detailed result page is displayed (via a link of the protein ID), providing basic information about the corrected protein sequence, including FixPred ID, species name, amino acid sequence of the corrected protein, the type of evidence of the protein existence, the date of publication, as well as about the original protein sequences identified by MisPred as erroneous, including the protein ID, the protein description, the database source, the species name, the erroneous protein sequences and the type of sequence error(s) identified by MisPred. Links to the source databases are also provided to help the user retrieve supplementary information about the original protein. Selected sequences may be downloaded in a variety of formats (XML, EXCEL, FASTA, LIST).

### Sequence analysis tools

**Figure 3.**
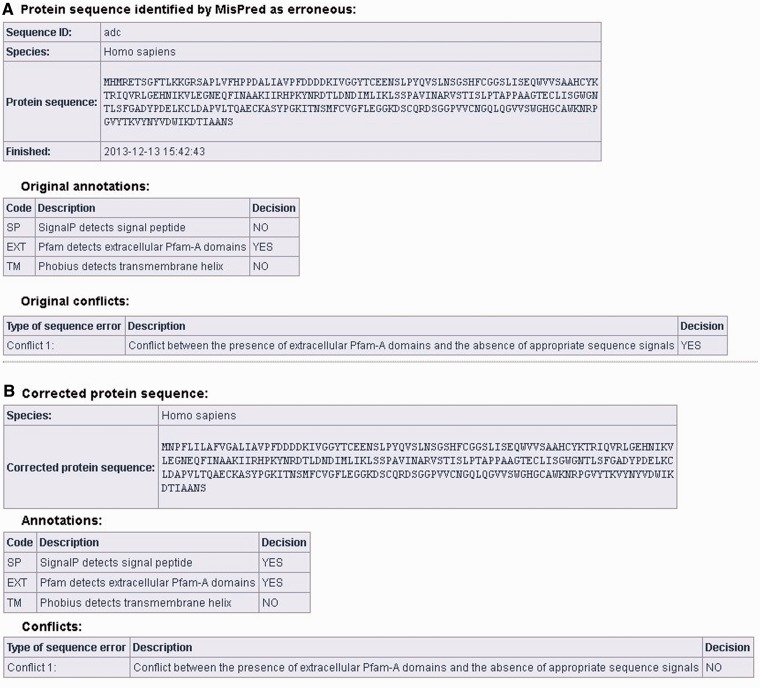
Correction of an erroneous protein sequence by the FixPred pipeline. (**A**) The upper part of the screen shot shows a *H. sapiens* protein sequence (NP_001184026.2, trypsin-3 isoform 3 preproprotein) that was identified as erroneous by MisPred tool 1 because it has an extracellular domain but lacks secretory signal peptide. (**B**) The erroneous protein was corrected by the FixPred pipeline in Step 2 by identifying a version (NP_002762.2, trypsin-3 isoform 2 preproprotein) that does not suffer from this type of error (see lower part of the screen shot).

Users can correct protein sequences identified as erroneous by MisPred on the ‘CORRECT YOUR SEQUENCE’ page using the FixPred pipeline. The results of the analysis are accessible in two different ways: without registration the results are available via a link for 72 h; registered users can access their results on the ‘Recent Results’ page for 20 days. The result page is divided into two parts (see Figure 3A and B). The first section displays information about the erroneous protein sequence submitted for analysis (automatically generated sequence ID, species name, protein sequence, task status, date and time of the completion of the MisPred analysis), the original MisPred annotations (presence or absence of signal peptide, transmembrane helices, etc.) and conclusion of the MisPred analysis: lists the type(s) of sequence error(s) identified by the MisPred tools. The second section shows the same information about the corrected protein sequence.

## Conclusions and future perspectives

In the future, the correction of erroneous protein sequences will be extended to sequences originating from other Metazoan species. We plan to update the sequence content of the FixPred database twice a year.

## Supplementary data

Supplementary data are available at *Database* Online.

## Funding

National Office for Research and Technology of Hungary (TECH_09_A1-FixPred9) and the Hungarian Scientific Research Fund (OTKA 101201). Funding for open access charge: Hungarian Scientific Research Fund (OTKA 101201).

*Conflict of interest*. None declared.
